# Next-generation strategies for anterior cruciate ligament repair: constructing biointelligent ligament grafts integrating biomimetic design, immune modulation, and sensory feedback

**DOI:** 10.3389/fbioe.2026.1847164

**Published:** 2026-06-19

**Authors:** Jing Lu, Jiayang He, Yuhan He, Hongyu Yi, Dujiang Yang, Junjie Chen, GuoYou Wang

**Affiliations:** 1 The Affiliated Traditional Chinese Medicine Hospital of Southwest Medical University, Luzhou, Sichuan, China; 2 Chongzhou Hospital of Traditional Chinese Medicine, Chengdu, Sichuan, China; 3 Southwest Medical University, Luzhou, Sichuan, China; 4 Chengdu University of Traditional Chinese Medicine, Chengdu, Sichuan, China; 5 Center for Orthopedic Diseases Research, Southwest Medical University, Luzhou, Sichuan, China

**Keywords:** anterior cruciate ligament reconstruction, biointelligent graft, digital twins, hierarchical biomaterials, proprioceptive neuralization, spatiotemporal immunomodulation, translational medicine

## Abstract

Anterior cruciate ligament (ACL) reconstruction conventionally prioritizes immediate mechanical stability; however, it does not fully prevent long-term post-traumatic osteoarthritis, residual neuromuscular deficits, or incomplete biological integration. This limitation reflects the gap between structural graft substitution and true functional regeneration, including insufficient ligament-bone interface healing, delayed graft vascularization and remodeling, loss of proprioceptive afferents, and the demanding intra-articular environment characterized by synovial fluid washout and high cyclic mechanical loading. To bridge this gap between conceptual biomaterials and clinical orthopedic practice, this review critically deconstructs the physiological bottlenecks of current ACL repair and reconstruction and outlines a translational roadmap for next-generation biointelligent ligament grafts. We evaluate emerging strategies including: (1) cross-scale hierarchical biomaterials designed to mitigate graft-tunnel micromotion, interfacial shear, and tunnel widening; (2) spatially retained and cell-responsive biological release systems that reduce the risk of uncontrolled intra-articular diffusion and ectopic ossification; (3) orthobiologic adjuncts, including platelet-rich plasma and platelet-rich fibrin, as clinically accessible but still insufficiently standardized tools for biological augmentation; (4) piezoelectric and mechanically shielded niches aimed at supporting proprioceptive neuralization; and (5) fluid-stable implantable sensors coupled with data-driven rehabilitation and digital twin concepts. By integrating biomimetic design, immune modulation, sensory restoration, and technology-readiness considerations, this review provides a clinically oriented framework for shifting ACL therapy from passive mechanical replacement toward active neuro-mechanical regeneration.

## 1 Introduction

The anterior cruciate ligament (ACL) is fundamentally more than a passive mechanical tether; it is a highly sophisticated neurosensory organ critical for joint proprioception and dynamic neuromuscular control. Consequently, ACL rupture is a catastrophic biomechanical and neurophysiological event. It is particularly prevalent among athletes in high-impact, pivoting sports, with female cohorts exhibiting distinct biomechanical and hormonal susceptibilities to injury (Kakavas et al., 2025; Spolaor et al., 2023). The disruption of the ACL eradicates essential proprioceptive nerve fibers, devastating joint position sense (Grevenstein et al., 2022). This coupled loss of mechanical restraint and sensory feedback not only severely impairs immediate athletic function but also inexorably predisposes the knee to secondary meniscal/chondral damage and the early onset of post-traumatic osteoarthritis (Arhos et al., 2025).

Current surgical gold standards predominantly rely on structural replacements—utilizing autografts, allografts, or synthetic substitutes—aimed at restoring gross knee stability to facilitate a rapid return to play (Thomson and Webb, 2025; Lara et al., 2024). Autografts remain the clinical benchmark due to their superior initial biomechanical profile; however, they are intrinsically constrained by donor-site morbidity and limited dimensional availability (Thomson and Webb, 2025; Nukuto et al., 2023). Conversely, while allografts and synthetic grafts circumvent harvest-site complications, they introduce formidable challenges regarding host immune rejection, delayed biological incorporation, and long-term structural degradation (Thomson and Webb, 2025; Nukuto et al., 2023). Despite continuous surgical refinements, including the advent of anatomic double-bundle reconstructions and supplementary extra-articular tenodesis, achieving optimal rotational kinematics remains an elusive goal. This clinical shortcoming is starkly evidenced by persistent residual joint laxity and unacceptable graft failure rates ranging from 4% to 17% (Gharpinde et al., 2024).

A critical flaw in the traditional reconstructive paradigm is not that all grafts are biologically inert, but that conventional reconstruction only partially restores the biological and neurosensory functions of the native ACL. Free tendon autografts, allografts, and synthetic substitutes differ substantially in cellular viability, immunogenicity, remodeling capacity, and host incorporation; nevertheless, all of them undergo a period of delayed vascularization, remodeling, and incomplete proprioceptive recovery after implantation. Standard graft harvesting and bone tunnel preparation also inevitably disrupt native mechanoreceptive networks, contributing to prolonged deficits in neuromuscular control (Balemane et al., 2025; Dong and Beynnon, 2023). While remnant-preserving techniques and primary repair strategies have been proposed to salvage native vascularity and mechanoreceptors for enhanced sensory feedback (Balemane et al., 2025; Hoogeslag et al., 2023), they present a severe clinical dilemma. Recent meta-analyses reveal that while primary repairs may yield comparable patient-reported outcomes, they often exhibit inferior mechanical stability and higher failure rates compared with traditional reconstructions, underscoring the complexity of balancing initial mechanical strength with long-term biological viability (Migliorini et al., 2023). Consequently, primary ACL repair has seen limited adoption among elite sports medicine practitioners due to these enduring efficacy concerns (Konstantinou et al., 2025), although the biomechanical and biological preservation of ACL remnants remains an important avenue warranting rigorous investigation (Nhan et al., 2021).

To bridge this mechanical-biological divide, modern translational research has increasingly pivoted toward active biological augmentation. The development of tissue-engineered scaffolds, such as those fabricated via supercritical carbon dioxide processing, represents a promising strategy to preserve native extracellular matrix architecture and reduce immunogenicity, thereby directing stem cell differentiation (Sherifi et al., 2020). Furthermore, the localized application of orthobiologics—including mesenchymal stem cells, platelet-rich plasma, platelet-rich fibrin, and bioactive growth factors—has been explored to enhance graft-bone integration, improve the tendon graft–bone tunnel interface in reconstruction, and support more favorable remodeling of the ligamentous graft environment (Sinkler et al., 2023)^.^ Experimental animal models further suggest that biologically augmented reconstructions may achieve superior early-stage biomechanical strength and more rapid collagen maturation compared with unaugmented repairs (Yanuar et al., 2025).

Simultaneously, the neuro-cognitive dimension of ACL recovery is gaining unprecedented recognition. ACL rupture primarily disrupts peripheral mechanoreceptive afferents within the ligament and joint capsule; over time, this peripheral sensory loss can induce secondary central sensorimotor reorganization, altered movement planning, and compensatory motor strategies associated with reinjury risk (Grooms et al., 2022; Picot et al., 2024). Although specialized neuromuscular training emphasizing external focus and kinetic-chain coordination (Ghaderi et al., 2021; Dhahbi et al., 2025), alongside targeted proprioceptive stimulation devices (Spolaor et al., 2023; Li et al., 2020), show clinical promise in mitigating high-risk landing biomechanics, overall rehabilitation outcomes remain inconsistent. The profound heterogeneity in graft choices, surgical execution, patient-specific biology, and rehabilitation adherence severely confounds standardized treatment algorithms and outcome prediction (Carrozzo et al., 2024; Chen et al., 2024a). Furthermore, adjunctive measures such as postoperative functional bracing have yielded controversial results regarding true proprioceptive protection and reinjury prevention (Heyworth et al., 2025).

Faced with these multifaceted clinical bottlenecks, the next-generation of ACL therapies must transcend passive structural substitution. The field is on the precipice of a paradigm shift toward “biointelligent” ligament grafts—dynamic, living constructs designed to synergistically integrate biomimetic structural gradients, spatiotemporally programmed immunomodulation, and active sensory feedback capabilities. By transitioning from pure mechanical reconstruction to holistic functional regeneration, these living grafts aim to orchestrate coordinated tissue remodeling, proprioceptive reinnervation, and adaptive motor control. This review systematically critically evaluates the core bottlenecks in current ACL repair and outlines the translational pathways for innovative smart biomaterials, neural reconstruction techniques, and intelligent monitoring technologies, ultimately providing a comprehensive roadmap for the clinical implementation of biointelligent ligament grafts.

### 1.1 Search strategy and scope of review

A structured literature search was performed in PubMed/MEDLINE, Web of Science Core Collection, Scopus, Embase, the Cochrane Library, and Google Scholar to identify relevant studies on ACL repair, ACL reconstruction, graft integration, biomaterials, orthobiologics, neuralization, and intelligent rehabilitation technologies. Search terms included combinations of the following keywords: “anterior cruciate ligament” OR “ACL”; “repair” OR “reconstruction” OR “graft”; “biointelligent graft” OR “biomimetic scaffold” OR “bioinspired scaffold” OR “tissue engineering” OR “electrospinning” OR “melt electrowriting” OR “3D printing”; “ligament-bone interface” OR “tendon-bone interface” OR “enthesis” OR “graft-bone healing”; “platelet-rich plasma” OR “PRP” OR “platelet-rich fibrin” OR “PRF” OR “orthobiologics”; “proprioception” OR “mechanoreceptor” OR “reinnervation” OR “neuralization”; and “implantable sensor” OR “digital twin” OR “closed-loop rehabilitation”. Original articles, preclinical studies, clinical studies, systematic reviews, meta-analyses, and high-quality narrative reviews were considered when they were relevant to ACL repair or reconstruction and to the biological, biomechanical, neural, or translational dimensions of ligament graft regeneration. This article was designed as a critical narrative review rather than a formal systematic review or meta-analysis; therefore, the literature was synthesized to identify mechanistic bottlenecks, emerging technologies, translational barriers, and future clinical pathways rather than to perform pooled quantitative analysis.

## 2 Current bottlenecks and emerging biointelligent strategies for ACL repair

### 2.1 Core bottlenecks and innovative breakthroughs in ACL repair

#### 2.1.1 Complexity of ligament-bone interface regeneration

The ligament-bone interface, or enthesis, represents a highly specialized anatomical region critical for the functional integration of soft ligament tissue with hard bone. This interface exhibits a sophisticated multi-tissue gradient characterized by gradual transitions in cellular phenotypes, extracellular matrix components, mineralization, fiber organization, and mechanical properties. The complexity of this interface poses substantial challenges for effective biological integration and regeneration, particularly in the context of ACL repair and reconstruction. The native insertion is not a simple boundary but a hierarchical fibrocartilaginous structure that transitions from ligament to unmineralized fibrocartilage, mineralized fibrocartilage, and bone, thereby enabling efficient load transfer and minimizing stress concentrations (Lei et al., 2021; Wang et al., 2024). Recent electrospinning-based and fascicle-inspired scaffold studies further emphasize that enthesis regeneration requires not only compositional gradients but also region-specific fiber architecture, strain-rate-dependent mechanical behavior, and multiscale deformation compatibility (Sensini et al., 2026; Di Lorenzo et al., 2026). Achieving effective biological integration at this interface is therefore hindered by the difficulty in recapitulating both the structural gradient and the dynamic mechanical environment of the native enthesis.

One of the fundamental issues in ligament-bone interface regeneration is the lack of a well-organized gradient structure in the interface region post-repair. The absence of this gradient leads to mechanical stress concentration at the interface, which compromises graft stability and increases the risk of graft loosening or failure (Lei et al., 2021; Wang et al., 2024). The mechanical mismatch between the soft ligament and hard bone tissues results in stress risers that can precipitate microdamage and impair long-term healing. This problem is compounded by the fact that current surgical techniques and biomaterial scaffolds often fail to induce spatially controlled differentiation of cells into the distinct tissue types required for the graded interface, such as fibroblasts, fibrochondrocytes, and osteoblasts, leading instead to scar tissue formation rather than regeneration of the native enthesis (Wang et al., 2024; Zou et al., 2023).

Moreover, the interface region’s cellular and biochemical microenvironment is highly heterogeneous, involving distinct cell populations and signaling gradients that regulate osteogenesis and ligamentogenesis in a spatially coordinated manner. However, current regenerative strategies struggle to induce the zonal regeneration of bone-inductive and ligament-inductive regions within the bone tunnel, which is critical for re-establishing the fibrocartilaginous enthesis (Xiong et al., 2022; Kajave et al., 2022). For example, although biomimetic scaffolds and decellularized extracellular matrix (ECM) materials have been developed to mimic the native ECM composition and mechanical properties, they often lack the precise spatial control of biochemical cues and mechanical gradients needed to direct region-specific cell differentiation and matrix deposition (Kajave et al., 2022; Suzuki et al., 2025). This limitation results in incomplete or disorganized interface regeneration, which undermines graft incorporation and functional recovery.

Recent advances have explored the use of graded biomaterials and composite scaffolds with controlled spatial distribution of bioactive factors to better replicate the native enthesis microenvironment. For instance, gradient hydrogels incorporating bioactive glass have been shown to promote synchronized regeneration of tendon, fibrocartilage, and bone tissues, enhancing biomechanical strength and mimicking the natural mineralization gradient (Liu et al., 2025a). Similarly, melt electrowriting (MEW) techniques have been employed to fabricate scaffolds with compositional and structural gradients that guide cell orientation and promote interfacial tissue regeneration, as demonstrated in periodontal ligament-to-bone interface models (Staples et al., 2022; Golafshan et al., 2023). These approaches highlight the importance of integrating both compositional and mechanical gradients to reduce stress concentration and facilitate effective biological integration.

Nevertheless, challenges remain in achieving precise spatial control of cell behavior and matrix organization at the ligament-bone interface. The complex interplay of mechanical loading, immune modulation, and biochemical signaling must be orchestrated to induce the formation of a functional enthesis rather than fibrous scar tissue (Cheng et al., 2022; Liu Y. et al., 2023). Moreover, the regeneration process must accommodate the dynamic healing environment within the bone tunnel, including vascularization and macrophage polarization, which influence tissue remodeling and integration (Zou et al., 2023; Cheng et al., 2022). The development of scaffolds and grafts that can mimic the native gradient structure and provide spatiotemporal delivery of bioactive cues remains a critical goal for improving graft stability and long-term outcomes in ligament repair.

In summary, the complexity of the ligament-bone interface regeneration arises from the intricate gradient structure of the native enthesis, the mechanical demands of the interface, and the need for spatially controlled biological cues to induce zonal tissue regeneration. Current technologies face difficulties in recapitulating these features, leading to stress concentration and poor graft integration. Emerging biomimetic strategies employing graded scaffolds, bioactive materials, and advanced fabrication techniques offer promising avenues to overcome these challenges by enabling more effective biological and mechanical integration at the ligament-bone interface (Lei et al., 2021; Xiong et al., 2022; Suzuki et al., 2025; Liu et al., 2025a).

#### 2.1.2 Insufficient vascularization and remodeling in the mid-substance of grafts

The mid-substance of grafts used in anterior cruciate ligament (ACL) reconstruction often suffers from inadequate vascularization, which critically limits nutrient delivery and metabolic waste removal essential for cellular viability and function. This insufficient angiogenesis hampers the remodeling process of the graft tissue, resulting in delayed or incomplete restoration of the ligament’s biomechanical properties. Experimental models have demonstrated that vascularized grafts, such as vascularized peroneus longus tendon grafts in rabbits, exhibit enhanced vascular infiltration and more robust collagen fiber integration at the tendon-bone interface compared to non-vascularized grafts. These vascularized grafts develop a “tidal-line” structure akin to native ACL insertions and demonstrate superior biomechanical strength at early and mid-term follow-ups (Zhang et al., 2020). Similarly, preservation of native ACL remnants during reconstruction has been shown to improve graft vascularity by maintaining synovial coverage rich in vascular-derived stem cells, which encircle the graft and promote revascularization (Hopper et al., 2023). Clinical imaging studies using dynamic contrast-enhanced magnetic resonance imaging (DCE-MRI) further corroborate that remnant-tensioning single-bundle ACL reconstructions yield better graft vascularity than double-bundle techniques, underscoring the importance of biological factors in graft revascularization (Kim et al., 2021).

The lack of sufficient blood vessel ingrowth into the graft mid-substance leads to a slower remodeling process, often termed “ligamentization,” which is crucial for regaining the native ligament’s mechanical properties. Histological analyses reveal that graft remodeling is a prolonged process spanning several years, characterized by dynamic changes in cellularity, collagen fiber organization, and expression of contractile proteins in fibroblasts and myofibroblasts. Notably, the remodeling process in human ACL grafts remains incomplete even after 9 years, with persistent differences in cellular composition and collagen crimp length compared to native ligaments (Mayr et al., 2021). The insufficient vascularization within the graft mid-substance is a key factor contributing to this protracted remodeling timeline, as it restricts the supply of oxygen and nutrients necessary for fibroblast proliferation and matrix synthesis.

To address these challenges, innovative biomaterial strategies and delivery systems have been developed to promote angiogenesis within grafts. For instance, autologous tissue-engineered polyethylene terephthalate (ATE-PET) grafts pre-vascularized by subcutaneous implantation demonstrate accelerated intra-articular ligamentization and intraosseous osseointegration compared to unmodified PET grafts in rabbit models. These pre-vascularized grafts exhibit increased α-SMA-positive vessels and myofibroblast aggregation, leading to improved biomechanical properties (Cai et al., 2021). Moreover, surface modification of synthetic grafts with bioactive coatings, such as strontium-doped hydroxyapatite (SrHA), has been shown to modulate macrophage polarization toward a pro-healing M2 phenotype, thereby enhancing angiogenesis and osteogenic differentiation essential for graft integration (Chen et al., 2024b). The immunomodulatory effect of such coatings facilitates a favorable microenvironment for vascular ingrowth and remodeling.

Cell-based approaches also hold promise in enhancing graft vascularization. Mesenchymal stromal cell-derived exosomes, particularly from infrapatellar fat pad sources, accelerate tendon-bone healing and intra-articular graft remodeling by modulating macrophage polarization and promoting fibrocartilage formation at the graft interface (Xu et al., 2022). Gene therapy strategies, such as transfection of human amniotic mesenchymal stem cells with Runx2, induce differentiation toward ligament fibroblasts and stimulate tendon-bone healing, partly by upregulating vascular endothelial growth factor (VEGF) and collagen synthesis (Xie et al., 2023). These biological interventions aim to overcome the intrinsic limitations of graft vascularization and remodeling by providing trophic support and enhancing cellular activities within the graft mid-substance.

In summary, insufficient vascularization in the mid-substance of ACL grafts significantly restricts nutrient supply and waste clearance, impeding the remodeling process and delaying mechanical recovery. Both preclinical and clinical evidence emphasize the need for graft designs and adjunctive therapies that promote angiogenesis and modulate the immune environment to facilitate efficient graft integration. The development of bioactive materials, cell-based therapies, and gene-modification approaches represent promising next-generation strategies to enhance vascularization and remodeling in ligament grafts, ultimately improving functional outcomes after ACL reconstruction.

From a clinical monitoring perspective, graft ligamentization should not be evaluated solely by time elapsed after surgery. Quantitative MRI parameters, particularly T^2^ relaxation time, together with objective knee laxity assessment, may provide a more integrated framework to evaluate graft maturation, cartilage status, and biomechanical recovery after ACL injury and reconstruction (Marchiori et al., 2021). Although such imaging-based approaches remain exploratory and require larger longitudinal validation, they offer a clinically feasible bridge between biological graft remodeling and functional knee stability.

#### 2.1.3 Challenges in proprioceptive nerve reinnervation

Proprioceptive nerve reinnervation in anterior cruciate ligament (ACL) grafts faces substantial challenges that critically affect sensory function restoration. One of the primary difficulties is the limited ability of proprioceptive nerve fibers to re-enter the graft tissue, resulting in a significant loss of sensory function post-reconstruction. This sensory deficit is particularly detrimental because proprioception—the body’s ability to perceive joint position and movement—is essential for knee joint stability and coordinated motor control. Histological and immunohistochemical studies have demonstrated that sensory corpuscles, such as Ruffini endings and free nerve endings, which mediate proprioceptive feedback, are markedly reduced in ACL grafts compared to native ligaments. For instance, a comparative analysis of human ACL transplants revealed that while mechanoreceptors are present in autografts and allografts, their density is significantly lower than in intact ligaments, and the allografts exhibit the lowest number of sensory receptors overall. This paucity of mechanoreceptors in grafts underscores the difficulty of achieving full sensory reinnervation after ligament reconstruction (Rebmann et al., 2020).

The regenerative process of proprioceptive nerves is further constrained by the absence of adequate neurotrophic support and suitable guidance structures within the graft environment. Neurotrophic factors are critical for promoting axonal growth and survival, yet their deficiency in the graft milieu hampers nerve fiber extension and integration. Additionally, the structural architecture of conventional grafts lacks the organized pathways necessary to direct regenerating nerve fibers effectively into the transplanted ligament tissue. As a result, nerve regeneration is often incomplete or misdirected, limiting the restoration of functional sensory innervation. This limitation is compounded by the fact that traditional ACL repair techniques primarily focus on mechanical stability and often neglect the neurobiological aspects of ligament healing, such as nerve ingrowth and mechanoreceptor reformation.

Moreover, the failure to adequately address neuralization during ligament repair has significant implications for knee joint function recovery. The absence or insufficiency of proprioceptive feedback can impair neuromuscular control, increasing the risk of joint instability and subsequent injury. Emerging surgical innovations, such as the agonist-antagonist myoneural interface (AMI), aim to restore physiologically relevant proprioceptive feedback by reinnervating muscle-tendon units. Experimental studies in animal models have shown that interventions like electrical muscle stimulation (EMS) can enhance the electrophysiological performance of reinnervated muscles, increasing nerve conduction velocity and motor unit recruitment. These findings suggest that EMS may facilitate nerve regeneration and improve proprioceptive afferent activation, potentially overcoming some of the inherent challenges in proprioceptive nerve reinnervation (Huang et al., 2024). However, translating these approaches to ACL grafts remains complex due to the intrinsic differences between muscle and ligament tissues and the need for specialized neurotrophic and structural support within the graft.

In summary, the challenges in proprioceptive nerve reinnervation after ACL reconstruction are multifactorial, involving the limited ingrowth of sensory nerve fibers into grafts, the scarcity of neurotrophic factors and guiding structures, and the traditional surgical focus on mechanical rather than sensory restoration. Addressing these challenges requires innovative strategies that integrate neurobiological principles to promote effective nerve regeneration and mechanoreceptor reformation, ultimately improving functional outcomes in ACL repair.

#### 2.1.4 Challenges in regulating early postoperative inflammation

The early postoperative inflammatory response following anterior cruciate ligament (ACL) reconstruction presents a significant challenge to tissue healing and graft integration. Excessive inflammation during this phase exacerbates tissue damage and impedes the regenerative processes essential for successful graft incorporation. For instance, polyethylene terephthalate (PET) artificial ligaments, widely used for their mechanical strength, often provoke a pronounced M1 macrophage-mediated inflammatory response due to their inert surface and mechanical abrasion. This response leads to the formation of excessive, disorganized scar tissue at the graft-bone interface, creating a space-occupying barrier that hinders graft-bone integration and contributes to complications such as bone tunnel enlargement (Wang S. et al., 2025). Similarly, synthetic grafts like the Ligament Advanced Reinforcement System (LARS) have been associated with chronic synovitis and foreign body reactions characterized by multinucleated giant cells and persistent inflammation, which compromise graft stability and longevity (Di Benedetto et al., 2020; Ambrosio et al., 2022). These findings underscore the detrimental impact of uncontrolled inflammation on the biological environment necessary for graft healing.

A critical obstacle in managing postoperative inflammation is the lack of precise temporal control systems that can balance the pro-inflammatory and reparative phases. The inflammatory response is dynamic, initially necessary for debris clearance and defense but requiring timely resolution to allow tissue regeneration. Current therapeutic strategies often fail to modulate this balance effectively, leading to either persistent inflammation or inadequate immune activation. Recent advances have explored sequential drug delivery systems, such as multilayer silk fibroin coatings on PET ligaments that release heparin early to mitigate inflammation and bone morphogenetic protein binding peptides later to promote osteogenesis. This approach demonstrated modulation of early inflammatory responses and enhanced graft maturation and graft-bone integration in animal models (Chen et al., 2023). Moreover, immunoregulatory hydrogel coatings combining gelatin methacrylate, polyethylene glycol diacrylate, and sulfated polysaccharides have been developed to downregulate M1 macrophage polarization, reduce scar formation, and promote M2 macrophage-mediated angiogenesis and osteogenesis, thereby facilitating scarless healing (Wang S. et al., 2025).

Immunomodulation has thus emerged as a pivotal direction to enhance graft bioactivity and healing outcomes. Targeting macrophage senescence and polarization states at the tendon-bone interface has shown promise; for example, therapies inhibiting CD14 expression reduced macrophage senescence and inflammation, leading to improved tendon-bone healing in murine models (Yao et al., 2025). Similarly, milk fat globulin protein E8 (MFG-E8) promotes macrophage efferocytosis and M2 polarization, which are associated with increased peri-tunnel bone formation, tighter graft-bone interfaces, and better mechanical properties post-ACL reconstruction (Geng et al., 2022). These immunoregulatory strategies highlight the importance of modulating the inflammatory microenvironment to prevent excessive tissue damage while promoting regenerative processes.

Nevertheless, the complexity of the immune response, including sex-specific differences and timing of interventions, complicates therapeutic design. For instance, adenosine, lidocaine, and magnesium (ALM) therapy demonstrated sex-specific immunomodulatory effects, reducing inflammation and enhancing tissue repair markers in males and females differently, suggesting that personalized approaches may be necessary (Morris et al., 2024; Morris et al., 2025). Additionally, larger graft diameters have been correlated with increased medial meniscal extrusion and osteophyte formation, indicating that mechanical factors may amplify inflammatory responses and early osteoarthritic changes post-reconstruction (Hada et al., 2025).

In summary, the early postoperative period after ACL reconstruction is marked by a delicate interplay between inflammation and repair. Excessive or prolonged inflammatory responses exacerbate tissue damage and hinder graft integration, while insufficient immune activation may impair healing. The absence of precise temporal immunoregulatory systems remains a major challenge. Advances in biomaterial coatings, targeted immunotherapies, and understanding of macrophage biology offer promising avenues to achieve balanced inflammation and enhance graft bioactivity. Future strategies must integrate these insights to develop biointelligent grafts capable of dynamic immunomodulation, ultimately improving clinical outcomes in ACL reconstruction. These core clinical bottlenecks and their corresponding next-generation biointelligent strategy categories are summarized in Table 1.

**TABLE 1:** Core clinical bottlenecks and next-generation solutions for anterior cruciate ligament repair.

Core clinical bottlenecks	Key pathophysiological mechanisms	Next-generation bio-intelligent strategy categories	Representative techniques or material examples	References
Difficulty in ligament-bone interface regeneration	Gradient structure deficiency; stress concentration; disordered cell differentiation	Biomimetic gradient interface design	3D-printed bioglass gradient scaffolds; melt electrowritten gradient fibers; electrospun region-specific fascicle-inspired scaffolds; gradient hydrogels	Lei et al. (2021), Wang et al. (2024), Kajave et al. (2022), Suzuki et al. (2025), Liu et al. (2025a), Staples et al. (2022), Golafshan et al. (2023)
Insufficient vascularization and remodeling of graft mid-section	Poor blood supply; hypoxic microenvironment; low fibroblast activity, remodeling process can take years and is incomplete	Active promotion of vascularization and immunomodulation	Prevascularized grafts (e.g., ATE-PET); vascularized graft models; MSC exosomes regulating macrophages	Zhang et al. (2020), Mayr et al. (2021), Cai et al. (2021), Xu et al. (2022)
Impairment of proprioceptive nerve regeneration	Lack of neurotrophic factors; mechanoreceptor density in grafts is significantly lower than in native ligaments	Neurotization and electrophysiological simulation	Nerve growth factor (NGF/BDNF) gradient; conductive/piezoelectric fiber scaffolds; biohybrid graft concepts	Rebmann et al. (2020), Guo Z et al. (2022), Tang et al. (2024), Chai et al. (2025), Pi et al. (2024), Lu et al. (2022), Amini et al. (2023)
Uncontrolled early postoperative inflammation	M1 macrophage-mediated excessive inflammatory response leading to scar tissue formation; synthetic grafts causing chronic synovitis	Temporal immunomodulation	Sequential intervention with multilayer silk fibroin coatings; targeting CD14 to inhibit macrophage senescence; MFG-E8 promoting M2 polarization	Wang W et al. (2025), Di Benedetto et al. (2020), Ambrosio et al. (2022), Chen et al. (2023), Yao et al. (2025), Geng et al. (2022)

### 2.2 Intelligent biomaterials and biomimetic graft design

**FIGURE 1:**
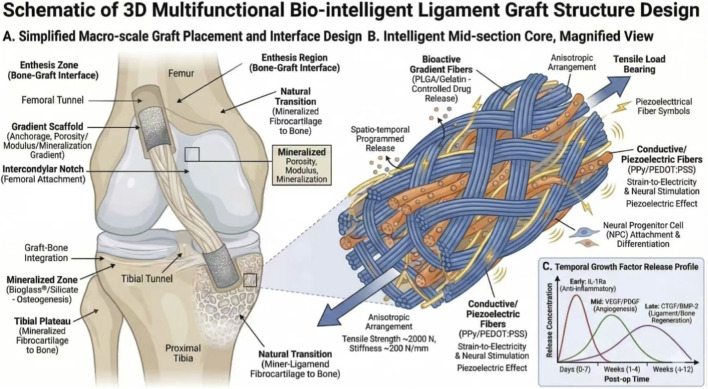
The figure depicts a schematic diagram of the structural design for a 3D multifunctional biointelligent ligament graft, illustrating its simplified macro-scale graft placement and interface design **(A)**, a magnified view of the biointelligent graft mid-substance/core with load-bearing fibers, porous micro/nano-fiber niches, piezoelectric/conductive elements, and localized biological-release modules **(B)**, and the time-release curve of growth factors **(C)**. This design aims to provide mechanical support and active regeneration through a biomimetic bone-ligament interface, anisotropic piezoelectric/conductive fibers, and spatiotemporally programmed biological cues.

The transition from passive structural substitutes to biointelligent ligament grafts demands a fundamental reconceptualization of biomaterial design. The native ACL is an anisotropic, multiscale hierarchical tissue characterized by non-linear viscoelasticity, region-specific fiber alignment, and coordinated biological remodeling. Merely matching the ultimate tensile strength of the native ligament is insufficient; modern biomimetic grafts must also replicate the mechanobiological microenvironment required for cellular infiltration, graft-bone integration, matrix remodeling, and proprioceptive recovery. A representative structural blueprint for a multifunctional biointelligent ligament graft is illustrated in Figure 1.

#### 2.2.1 Hierarchical anisotropic fibers: overcoming the “porosity vs. mechanics” paradox

The deployment of multifunctional fiber-based materials is a cornerstone of next-generation graft engineering. Conventional strategies often utilize electrospun fibers—co-spinning biodegradable polymers like poly (lactic-co-glycolic acid) (PLGA) with natural proteins (e.g., gelatin)—to achieve localized delivery of therapeutic growth factors (Guo L. et al., 2022). Furthermore, structurally tailored Janus nanofibrous scaffolds, integrating materials like silk fibroin and polycaprolactone (PCL), can provide vital directional cues to guide cellular adhesion and extracellular matrix alignment (Tang et al., 2024). To simulate the native electrophysiological microenvironment, electroactive components, including conductive or piezoelectric fibers fabricated from PVDF or incorporating ZIF-8 nanoparticles, have been embedded within these matrices to provide dynamic bioelectrical stimulation for enhanced tissue repair (Chai et al., 2025; Pi et al., 2024).

Translational Challenges and Critical Appraisal: Despite these *in vitro* successes, standalone electrospun or nanofibrous scaffolds face a fatal clinical paradox: the “Porosity vs. Mechanics” trade-off. To achieve the requisite biomechanical strength for ACL reconstruction (exceeding 2000 N), nanofibrous mats must be fabricated with extreme density. However, this densely packed architecture results in sub-micron pore sizes, effectively acting as a 2D barrier that restricts fibroblasts and macrophages to superficial colonization, while the graft core remains acellular and necrotic. Furthermore, the bulk degradation of widely used aliphatic polyesters like PLGA generates localized acidic byproducts. In the avascular, enclosed space of the knee joint, this acid accumulation frequently triggers catastrophic sterile inflammation and aggressive osteolysis at the bone tunnel. Future bio-intelligent designs must utilize cross-scale hierarchical braiding techniques—where ultra-strong macroscopic multifilament yarns provide immediate load-bearing capacity, while interspersed, highly porous micro/nano-fibers act as bioactive, acid-buffered niches for rapid 3D cellular infiltration.

#### 2.2.2 Spatially patterned 3D architectures and the “triple biomimetic” design

The structural engineering of' ACL grafts has evolved toward a highly sophisticated “triple biomimetic” paradigm, aiming to replicate the native ligament at the macroscopic, mesoscopic, and microscopic scales simultaneously.

At the macro- and meso-scales, patient-specific anatomical conformity and functional biomechanics are paramount. Rather than relying on generic cylindrical grafts, modern computational modeling and MRI data enable the design of grafts that precisely replicate the patient’s unique isometric attachment footprints. Furthermore, structurally decoupling the graft into a double-bundle architecture—mimicking the anteromedial (AM) and posterolateral (PL) bundles—is critical. This braided or woven topology not only restores complex rotational stability but also replicates the native ACL’s non-linear viscoelastic “toe-region,” a crucial shock-absorbing mechanism that prevents catastrophic graft failure during sudden deceleration or pivoting maneuvers (Kadyr et al., 2024).

At the micro-scale, engineering a seamless gradient interface addresses the most vulnerable link in ACL reconstruction: graft-to-bone integration within the bone tunnel (Kajave et al., 2022). The abrupt mechanical mismatch between a compliant soft-tissue graft and rigid bone creates shear stress at the tunnel aperture, clinically contributing to graft–tunnel micromotion, interfacial shear, tunnel widening, and longitudinal graft displacement. Advanced fabrication modalities, such as 3D-printed bioglass gradients, electrospun or melt-electrowritten gradient fibers, and spatially mapped polyelectrolyte complexation fibers, can partially emulate the mineralization and fiber-orientation gradients of the native enthesis (Kajave et al., 2022; Liu et al., 2024; Sensini et al., 2026; Di Lorenzo et al., 2026). These compositionally and structurally graded scaffolds provide site-specific biochemical and mechanical cues that may guide mesenchymal stromal cells toward regionalized fibrochondrocytic and osteogenic phenotypes, thereby reducing interfacial stress concentration and accelerating graft incorporation.

Translational Challenges and Critical Appraisal: The seamless integration of these “triple biomimetic” features remains an immense manufacturing bottleneck. Industrial braiding machines can produce mechanically robust macroscopic structures, but they lack the micro-precision required to deposit biochemical or mineralization gradients. Conversely, high-resolution 3D bioprinting can perfectly pattern gradient interfaces but struggles to yield continuous, load-bearing fibers capable of withstanding cyclic fatigue. The ultimate realization of bio-intelligent grafts relies on the imminent convergence of additive manufacturing (e.g., melt electrowriting) with advanced textile engineering, ensuring that biological gradients are not compromised by the overarching demand for immediate mechanical resilience.

### 2.3 Biological regulation strategies: from passive support to active regeneration

The intrinsic healing capacity of the intra-articular ACL is notoriously poor, largely due to the synovial fluid environment which prematurely dissolves fibrin clots and dilutes local autocrine signals. Consequently, bio-intelligent grafts must actively command the local biological cascade. However, simply loading grafts with supratherapeutic doses of biologics is not only highly inefficient but clinically hazardous. Next-generation strategies must evolve from bulk passive delivery to precise, demand-driven cellular regulation.

#### 2.3.1 Spatiotemporal release systems: mitigating the perils of ectopic ossification

The natural healing of ligamentous tissue proceeds through overlapping phases: inflammation, proliferation (angiogenesis), and remodeling (ligamentization and osteogenesis). An ideal bio-intelligent graft would theoretically orchestrate this via sequential delivery: early release of anti-inflammatory agents like interleukin-1 receptor antagonist (IL-1Ra) to blunt aggressive synovitis (Liu et al., 2025b), mid-phase release of VEGF/PDGF for revascularization, and late-phase delivery of connective tissue growth factor (CTGF) and bone morphogenetic protein-2 (BMP-2) to fortify the enthesis.

Translational challenges and critical appraisal: While *in vitro* models neatly demonstrate these sequential release profiles, relying on passive polymer degradation (e.g., PLGA hydrolysis) for temporal control in a dynamic, weight-bearing knee joint is highly unpredictable. The most critical translational barrier is the management of potent osteoinductive factors like BMP-2. Uncontrolled burst release or synovial diffusion of BMP-2 away from the bone tunnel inevitably triggers catastrophic heterotopic ossification (ectopic bone formation within the joint space) and debilitating joint stiffness (James et al., 2016). Therefore, the paradigm must shift from “controlled release” to “spatially locked, cell-demanded delivery.” Advanced strategies now use covalent tethering or specific peptide linkers that bind growth factors directly to the scaffold matrix. These factors remain entirely inert and structurally bound until infiltrating local cells secrete specific matrix metalloproteinases (MMPs) to cleave the linker, ensuring that potent factors like BMP-2 are exclusively presented to osteoblasts exactly at the bone tunnel interface, rigidly preventing intra-articular leakage (Lutolf and Hubbell, 2005).

#### 2.3.2 Cell homing and gene therapy: confronting the hypocellular niche

To circumvent the immense regulatory and logistical hurdles of exogenous stem cell transplantation, utilizing the chemokine stromal cell-derived factor-1 (SDF-1) to create a chemotactic gradient for endogenous mesenchymal stem cell (MSC) homing has emerged as a compelling strategy (Lu et al., 2022; Yang et al., 2023). Concurrently, Gene-Activated Scaffolds (GAS) represent an elegant approach to reprogramming these recruited cells *in situ*. By impregnating scaffolds with non-viral vectors (e.g., rAAV or plasmid DNA) encoding key tenogenic and fibrogenic transcription factors like TGF-β and scleraxis (SCX), the graft essentially becomes a localized bioreactor, directing recruited fibroblasts toward sustained extracellular matrix deposition and organized ligamentous remodeling without eliciting severe immune responses (Amini et al., 2023; Stehle et al., 2024; Amini et al., 2022).

Translational challenges and critical appraisal: Despite the theoretical elegance of these strategies, the avascular, hypocellular nature of the intra-articular space severely blunts their *in vivo* efficacy. Unlike the bone marrow compartment, synovial fluid contains a paucity of circulating progenitor cells, making SDF-1 gradients functionally limited if not augmented by bone marrow venting (microfracture) during surgery. Furthermore, penetrating the dense, fibrous architecture of a remodeling graft poses an immense physical barrier to gene vector transfection. A more insidious clinical risk is the unregulated overexpression of profibrotic genes. Sustained, uncoupled delivery of TGF-β is a primary driver of arthrofibrosis—a pathological proliferation of disorganized scar tissue that leads to severe knee contracture. Thus, future GAS platforms must incorporate mechano-responsive promoters, ensuring that tenogenic gene expression is dynamically upregulated by the physiological tension of rehabilitation exercises and downregulated during rest, thereby preventing pathological over-fibrosis.

#### 2.3.3 Orthobiologics in ACL repair and reconstruction: PRP and PRF

Orthobiologics represent the most clinically accessible form of biological augmentation for ACL repair and reconstruction. Among them, platelet-rich plasma (PRP) and platelet-rich fibrin (PRF) are particularly relevant because they provide autologous platelet-derived cytokines and growth factors without requiring exogenous cell expansion, complex gene manipulation, or highly specialized manufacturing. PRP contains bioactive mediators such as platelet-derived growth factor, transforming growth factor-β, vascular endothelial growth factor, insulin-like growth factor-1, and other cytokines that may regulate angiogenesis, collagen synthesis, inflammatory balance, and cell recruitment. In ACL reconstruction, PRP has been applied through graft soaking, bone tunnel injection, intra-articular injection, or repeated postoperative administration. Mechanistically, PRP may support early vascular ingrowth and matrix remodeling; however, current clinical evidence remains heterogeneous. Reported benefits appear more consistent for early pain relief or short-term recovery than for long-term graft maturation, objective stability, or durable functional improvement (Zhang et al., 2025; Li et al., 2025). This inconsistency is partly attributable to variability in leukocyte content, platelet concentration, activation method, injection site, timing, dose, and outcome assessment.

PRF differs from liquid PRP by forming a fibrin-rich three-dimensional matrix that may serve simultaneously as a platelet-derived growth factor reservoir and a provisional scaffold for cell migration. This property is particularly attractive in ACL repair because synovial fluid can wash out soluble biologics and disrupt stable clot formation. PRF may therefore provide a more spatially retained biological platform for graft coating, bone tunnel augmentation, or gap-bridging primary ACL repair. Experimental evidence suggests that PRF-based gap-bridging strategies can facilitate the formation of stable repair tissue in ACL defect models, while limited clinical studies have explored PRF application during anatomic ACL reconstruction (Kasl et al., 2022; Weng et al., 2023). Nevertheless, PRF remains less standardized than PRP in the ACL field, and its clinical value should be interpreted cautiously until larger prospective studies clarify preparation protocols, fibrin architecture, leukocyte content, implantation technique, imaging-based graft maturation, and long-term functional outcomes.

Therefore, PRP and PRF should not be presented as stand-alone regenerative solutions. A more realistic translational role is to treat them as clinically feasible orthobiologic adjuncts that may become more effective when combined with spatially retained scaffolds, interface-specific delivery systems, and standardized rehabilitation protocols. In this sense, PRP and PRF bridge current clinical practice and future biointelligent graft design: they are already technically deployable in the operating room, but their reproducibility, biological potency, and outcome predictability remain insufficiently mature. The major material platforms and biological regulatory strategies for constructing biointelligent ACL grafts are summarized in Table 2.

**TABLE 2:** Multifunctional materials/platforms for constructing bio-intelligent ligaments.

Material category/Platform	Core design principles	Key functions	Application objectives	References
Gradient fiber scaffolds	Mimicking ligament anisotropy and interface gradients	Providing mechanical guidance; spatially controlling cell behavior and differentiation	Achieving ligament-bone insertion regeneration, reducing stress concentration; matching native ACL mechanical properties	Lei et al. (2021), Wang S et al. (2024), Xiong et al. (2022), Kajave et al. (2022), Liu et al. (2025a), Staples et al. (2022), Golafshan et al. (2023), Kadyr et al. (2024), Liu et al. (2024), Sensini et al. (2026), Di Lorenzo et al. (2026)
Sequential release carriers	Responding to healing stages (inflammation-proliferation-remodeling)	Dynamic delivery of bioactive molecules to match healing timeline	Achieving sequential therapy for anti-inflammation, pro-angiogenesis, and osteogenesis/ligamentogenesis	Chen et al. (2023), Liu Y. et al. (2023)
Conductive/piezoelectric materials	Converting mechanical strain into electrical signals; providing exogenous conductivity	Simulating neuro-electrophysiological environment; providing electrical stimulation to promote nerve regeneration	Polypyrrole/silk fibroin composite scaffolds; piezoelectric fiber dressings managing healing through electrical stimulation	Chai et al. (2025), Pi et al. (2024), Lu et al. (2022), Liu Z. et al. (2022), Tim-Yun Ong et al. (2022)
Immunomodulatory interfaces	Surface chemical modifications regulating immune cell receptors and polarization	Actively modulating macrophage phenotypes (from pro-inflammatory M1 to reparative M2)	SrHA coatings modulating immunity to promote integration; immunomodulatory hydrogel coatings achieving scarless healing	Chen et al. (2024b), Wang W et al. (2025), Yao et al. (2025)
Gene-activating matrices	Scaffolds loaded with non-viral vectors for *in situ* gene delivery	Local, sustained expression of therapeutic genes directly programming host cells	pNaSS-grafted PCL films delivering rAAV-TGF-β/FGF-2, stimulating ACL cell repair activity	Amini et al. (2023), Stehle et al. (2024), Amini et al. (2022)

### 2.4 Neuralization and proprioceptive reconstruction: the overlooked key dimension

**FIGURE 2:**
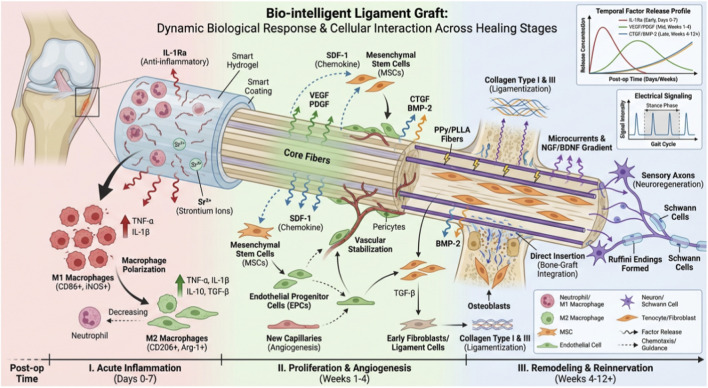
The figure illustrates the dynamic biological responses and cellular interactions of bio-intelligent ligament grafts during various repair stages, depicting the acute inflammatory phase (0–7 days), proliferative angiogenesis phase (1–4 weeks), and remodelling and re-innervation phase (4–12 weeks). It depicts how the graft modulates macrophage polarisation, angiogenesis, osteogenic and ligamentous differentiation, and neuroregeneration through intelligent hydrogels and conductive fibres, whilst exhibiting spatiotemporal characteristics of growth factor release and electrical signal conduction.

The fundamental paradox of traditional anterior cruciate ligament (ACL) reconstruction lies in treating a complex neurosensory organ as a mere mechanical rope. While the native ACL provides dynamic neuromuscular joint stabilization via an intricate network of mechanoreceptors (Ruffini endings, Pacinian corpuscles), conventional grafts remain permanently denervated. Restoring this “proprioceptive blind spot” requires engineering bio-intelligent niches that actively recruit and sustain sensory axons. However, translating neural tissue engineering into the hostile, mechanically demanding intra-articular environment presents profound physiological and biomaterial challenges. The temporal sequence of inflammation, angiogenesis, matrix remodeling, and reinnervation in biointelligent graft repair is schematically summarized in Figure 2.

#### 2.4.1 Overcoming synovial washout: spatiotemporal gradients for neurotrophic guidance

The application of neurotrophic factors (such as NGF and BDNF) is classically employed to guide sensory axon elongation and modulate neuroimmune crosstalk (Tim-Yun Ong et al., 2022; Ozgen et al., 2025). However, the intra-articular reality is severe: direct coating or simple encapsulation of these factors results in immediate burst release and rapid dilution by the constantly circulating synovial fluid. Consequently, the critical chemotactic gradients required for directional axonal sprouting fail to establish within the avascular core of the graft.

To reduce synovial washout, spatiotemporally retained neurotrophic delivery systems could be adapted from enzyme-responsive hydrogel platforms and bioactive electrospun nerve scaffolds. Matrix metalloproteinase-sensitive linkers, tethered growth factors, electrostatically immobilized neurotrophins, or mechanically responsive micro/nano-carriers may allow neurotrophic cues to remain scaffold-associated until cell-mediated remodeling or local mechanical stimulation triggers their release (Lutolf and Hubbell, 2005; Sandoval-Castellanos et al., 2021). For example, electrospun scaffolds with heparin-bound NGF or BDNF have been shown to provide sustained neurotrophin release and promote neurite outgrowth and Schwann cell migration in neural tissue-engineering models (Sandoval-Castellanos et al., 2021). In the ACL setting, however, these approaches should be considered preclinical design concepts rather than clinically validated solutions. Their translational value will depend on whether they can maintain a localized NGF/BDNF gradient within the graft while avoiding uncontrolled intra-articular diffusion, ectopic sensitization, or neuroma-like responses at the graft periphery.

#### 2.4.2 The piezoelectric paradigm: translating joint kinematics into endogenous electrophysiological cues

Conductive polymers, such as polypyrrole (PPy) and PEDOT:PSS, have been extensively studied for peripheral nerve regeneration due to their ability to provide exogenous electrical stimulation, which upregulates neurogenesis-related pathways (e.g., MAPK/ERK) and accelerates Schwann cell alignment (Zhao et al., 2020; Liu Z. et al., 2022; Montes et al., 2023; Oktay et al., 2025; Tang et al., 2020; Guan et al., 2022; Xiong et al., 2023; Ghosh et al., 2020). However, applying these bio-batteries to ACL repair faces a critical translational barrier: they are typically brittle, lack the immense tensile strength required for ligamentous loading, and rely on external power sources which are clinically impractical for deep intra-articular implants (Guo Z. et al., 2022; Cheng et al., 2025; M et al., 2025; Tonellato et al., 2020; Chen et al., 2020; Aydeger et al., 2023; Chen et al., 2025; Muzzio et al., 2025).

A potential next step for biointelligent ligament design is the piezoelectric paradigm. Piezoelectric polymers, such as poly (L-lactic acid) and polyvinylidene fluoride-based materials, can transduce mechanical deformation into localized transient surface electrical charges without an external power supply (Das et al., 2025; Lee et al., 2011). In principle, incorporating piezoelectric nanofibers into the core-shell architecture of a load-bearing ACL graft could allow cyclic knee loading during gait or rehabilitation to generate localized bioelectrical cues. This hypothesis is supported indirectly by neural tissue-engineering studies showing that electrospun PVDF-TrFE piezoelectric scaffolds can support dorsal root ganglion neurite extension and that electrospun piezoelectric composite nanofibers can accelerate peripheral nerve regeneration (Lee et al., 2011; Mao et al., 2022). However, this remains a hypothesis-driven design strategy in the ACL setting. ACL-specific validation is still required to determine charge output under physiological knee loading, long-term fatigue durability, neural specificity, safety, and whether such bioelectrical cues can meaningfully improve proprioceptive functional recovery.

#### 2.4.3 Biohybrid grafts and “mechanical shielding” niches

The most ambitious Frontier in proprioceptive restoration involves biohybrid grafts pre-seeded with living cellular components, such as Schwann cells or dorsal root ganglion (DRG) neurons, to establish an immediate bioactive neurotrophic relay (Mayoral et al., 2022; Yeou and Shin, 2025; Ghosh et al., 2022; Yi et al., 2023; Liu H. et al., 2023; Liu J. et al., 2022). Yet, directly integrating fragile neural cells into an ACL graft exposes them to catastrophic macroscopic shear forces and cyclic tensile loads (often exceeding 2000 N), leading to rapid stress-induced apoptosis.

To achieve viable biohybrid neurotization, the graft architecture must provide a profound “mechanical shielding” niche. Advanced biomimetic strategies now use structurally decoupled designs, where ultra-high-strength anisotropic fibers (e.g., braided PET or PEEK) absorb the macroscopic biomechanical loads, while an interpenetrating, highly viscoelastic hydrogel network provides a zero-stress sanctuary for the embedded neural cells (Chaudhuri et al., 2016). This core-shell micro-mechanics approach ensures that the hydrogel matrix safely dissipates shear stress via rapid stress-relaxation, maintaining an optimal soft-tissue stiffness (typically <10 kPa) that mimics the native endoneurium (Yang et al., 2021; Kohestani et al., 2024). By mechanically isolating the biological payload from the structural tether, these advanced biohybrid scaffolds enable the long-term survival and functional integration of DRG neurons, paving the way for true neuro-mechanical ACL regeneration.

### 2.5 Intelligent monitoring and personalized rehabilitation

**FIGURE 3:**
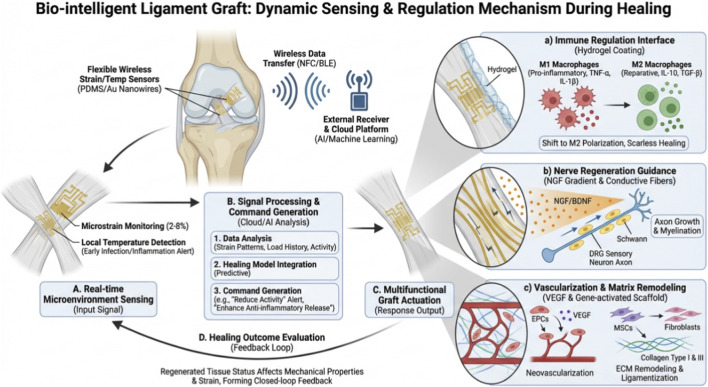
The figure illustrates the dynamic sensing and regulation mechanism during bio-intelligent ligament graft repair. It demonstrates the entire process: real-time monitoring of the microenvironment via flexible wireless strain/temperature sensors **(A)**, generation of regulatory commands through cloud-based AI analysis **(B)**, multifunctional responses achieved by the graft through immune regulation, nerve regeneration guidance, vascularisation, and matrix remodelling **(C)**, and closed-loop regulation formed by feedback from repair outcomes **(D)**.

The ultimate evolution of bio-intelligent ligament grafts culminates in their capacity to actively perceive and communicate their structural and biological status. By integrating real-time sensing elements with advanced computational modeling, these “perceptive” grafts aim to close the loop between surgical intervention and long-term functional rehabilitation, replacing generic, time-based recovery protocols with data-driven, patient-specific management. The proposed sensor–AI analysis–biological response feedback loop is illustrated in Figure 3.

#### 2.5.1 “Perceptive” grafts: the implantable sensing paradox

The integration of miniaturized sensing technologies—such as optical fiber Bragg gratings (FBGs) and flexible capacitive/piezoresistive strain sensors—theoretically allows for the continuous, *in situ* monitoring of critical parameters including graft micro-strain, localized tension, and temperature fluctuations (Yang et al., 2025; Wang W. et al., 2025). Monitoring these metrics provides an unprecedented objective window into early graft elongation, impending mechanical failure, or subclinical acute inflammation (Yang et al., 2025; Wang W. et al., 2025).

Translational challenges and critical appraisal: Despite remarkable demonstrations in wearable or superficial diagnostics, deploying these sensors deep within the high-load, dynamic intra-articular environment exposes critical material and physical paradoxes. First, silica-based FBGs possess inherent brittleness; they are highly susceptible to catastrophic fracture under the extreme shear and bending moments characteristic of maximal knee flexion. Conversely, ultra-compliant hydrogel-based sensors face the formidable challenge of the synovial fluid environment. Prolonged exposure to this aqueous, ion-rich fluid induces severe osmotic swelling in soft sensors, resulting in profound baseline signal drift and eventual delamination from the host graft material, rendering long-term mechanical readouts meaningless. Furthermore, wireless power transfer and data telemetry (via RFID or NFC) suffer from massive signal attenuation and backscatter interference when transmitted through dense bone, highly vascularized muscle, and synovial fluid (Chaudhuri et al., 2016). Future designs must shift towards highly robust, fluid-impermeable encapsulation strategies and utilize low-frequency ultrasound or acoustic telemetry, which penetrates deep tissue more efficiently than electromagnetic waves.

#### 2.5.2 Closed-loop data-driven rehabilitation management

Current post-ACL reconstruction rehabilitation relies heavily on subjective patient feedback and intermittent, static clinical assessments. The integration of continuous, wireless sensor data seamlessly transmitted to external computational platforms facilitates dynamic, remote monitoring (Tzepkenlis et al., 2025; Zhu et al., 2025; Skov et al., 2024; Tsakanikas et al., 2022; Pedroso et al., 2025). By applying machine learning algorithms (e.g., XGBoost, LSTM networks) to vast datasets of high-resolution kinematic and physiological parameters, clinicians can establish personalized, automated scoring models that predict recovery trajectories and adjust therapy intensity in real-time (Zhu et al., 2025; Skov et al., 2024; Tsakanikas et al., 2022; Pedroso et al., 2025; Kondylakis et al., 2020).

Translational challenges and critical appraisal: The primary limitation of current data-driven rehabilitation is its inherently “open-loop” nature. While external wearable sensors can precisely track macroscopic joint kinematics (e.g., step count, angular velocity), they remain entirely blind to the microscopic internal forces experienced by the healing graft at the bone tunnel. To truly revolutionize rehabilitation, the field must advance to “closed-loop actuation.” In this paradigm, when implanted sensors detect hazardous micro-strain thresholds indicating impending graft failure, the system does not merely alert the physician; it automatically triggers the localized release of anti-inflammatory or stiffening agents within the smart graft itself, providing an immediate, autonomous biological or mechanical intervention (Son et al., 2014).

#### 2.5.3 Digital twins and the “sim-to-real” biological gap

The synthesis of continuous sensor data, advanced imaging, and artificial intelligence has catalyzed the conceptualization of biomechanical “Digital Twins”—dynamic, *in silico* replicas of a patient’s specific knee joint. These highly personalized computational models empower orthopedic surgeons to conduct rigorous preoperative simulations (optimizing tunnel placement and graft tension) and enable AI-driven predictive analytics to forecast long-term outcomes, such as the timeline for ligamentization or the impending risk of post-traumatic osteoarthritis (Ferro et al., 2025; Dean et al., 2024; Ooka, 2025; Sadee et al., 2025; Vallee, 2024).

Translational challenges and critical appraisal: While digital twins excel at predicting macroscopic biomechanical stress distributions (using finite element analysis), they currently face an insurmountable “Biological Ground Truth” bottleneck. A true digital twin requires continuous updates of both mechanical *and* biological/chemical states. Currently, real-time non-invasive measurement of *in vivo* immune status, such as the precise spatiotemporal ratio of M1 to M2 macrophages, and the exact rate of collagen cross-linking within the joint remains unavailable. Without these critical biochemical inputs, AI models are forced to rely on generalized, static biological assumptions, significantly reducing their predictive fidelity for individual patients. Bridging this gap requires the development of novel biosensors capable of continuously tracking specific inflammatory biomarkers and pH levels within the synovial fluid, feeding multi-omics data into the digital twin to achieve true chemo-mechanical predictive accuracy.

### 2.6 Bench-to-bedside translation and technology readiness of biointelligent ACL grafts

Although biointelligent ACL grafts offer a compelling conceptual direction, their clinical translation depends on whether they can be converted into reproducible, affordable, scalable, and surgically deployable solutions. In practical terms, the distance from bench to bedside varies greatly across the strategies discussed in this review. Orthobiologics such as PRP and PRF are closest to clinical use because they can be prepared from autologous blood and applied during conventional ACL procedures. However, their biological variability and lack of standardized preparation protocols limit reproducibility. By contrast, gradient scaffolds, immunomodulatory coatings, gene-activated matrices, piezoelectric neuralized grafts, implantable sensors, and digital twin systems remain at earlier stages of translational maturity because they require additional validation of mechanical fatigue resistance, long-term biocompatibility, sterility, storage stability, regulatory classification, and clinical workflow compatibility.

A realistic translational pathway should proceed in a staged manner. First, candidate graft platforms must demonstrate reproducible manufacturing, sterilization tolerance, and stable mechanical behavior under cyclic loading that approximates the postoperative knee environment. Second, ACL-specific large-animal models are required to evaluate graft-bone integration, tunnel widening, inflammatory response, vascularization, ligamentization, and proprioceptive recovery under clinically relevant fixation and rehabilitation conditions. Third, early human feasibility studies should prioritize safety, arthroscopic deployability, operative time, graft fixation compatibility, and imaging-based graft maturation before claiming superiority in long-term functional outcomes. Finally, randomized clinical trials should include not only patient-reported outcomes and laxity testing but also quantitative MRI, return-to-sport criteria, reinjury rate, adverse events, cost-effectiveness, and scalability across different hospital settings.

From a regulatory and health-system perspective, the complexity of each platform should guide expectations. A PRP- or PRF-augmented procedure may be evaluated within relatively familiar orthobiologic frameworks, whereas gene-activated scaffolds, cell-loaded constructs, drug-device combinations, and sensor-integrated implants may require substantially more demanding regulatory pathways. Therefore, the near-term clinical opportunity is not to replace standard ACL reconstruction with a fully autonomous living graft, but to integrate selected biointelligent functions stepwise into existing surgical workflows. Interface-specific scaffold design, standardized orthobiologic augmentation, clinically monitorable graft maturation, and data-informed rehabilitation may represent the most realistic intermediate milestones. The technology readiness level (TRL) framework has been widely used to contextualize the translational maturity of biomedical technologies, including diagnostics and medical devices; therefore, it was applied here as a qualitative tool rather than as a formal regulatory classification (U.S. Department of Health and Human Services, 2026). The technology-readiness profile of the major strategies discussed in this review is summarized in Table 3. Technology readiness level (TRL) is a staged framework used to describe the maturity of a technology, ranging from TRL 1, where basic principles are observed, to TRL 9, where the technology has been validated and deployed in its intended operational or clinical setting (U.S. Department of Health and Human Services, 2026). In this review, TRL values are used as qualitative ACL-specific estimates of translational maturity rather than formal regulatory certification.

**TABLE 3:** Technology readiness and translational barriers of emerging ACL repair strategies.

Research focus	ACL-specific TRL estimate	Current evidence level	Main translational barrier	Next required work
PRP/PRF orthobiologics	PRP: 6–7; PRF: 4–6	PRP has randomized-trial/meta-analysis evidence; PRF has smaller clinical and preclinical evidence	Variable preparation, dose, leukocyte content, activation, and delivery timing	Standardized RCTs with imaging-based graft maturation and health-economic endpoints
Biomimetic gradient scaffolds/electrospun enthesis constructs	3–4	*In vitro* and small-animal evidence; advanced scaffold characterization	Fatigue durability, sterilization, reproducibility, and fixation compatibility	Large-animal ACLR studies, cyclic fatigue tests, and arthroscopic deployability testing
Spatiotemporal release and immunomodulatory coatings	3–4	Mainly preclinical biomaterial studies	Burst release, ectopic ossification risk, and drug-device regulatory complexity	Spatially locked release validation, dose-safety studies, and GLP animal experiments
Gene-activated scaffolds/exosome systems	2–3	Early preclinical proof-of-concept	Off-target fibrosis, biodistribution, vector safety, and manufacturing burden	Localized expression control, long-term safety, and regulatory pathway definition
Piezoelectric/conductive neuralized grafts	2–3	Proof-of-concept mainly outside ACL-specific clinical use	Tensile strength, charge stability, neuroma risk, and sensory-function validation	ACL-specific animal models with mechanoreceptor, gait, and proprioceptive endpoints
Implantable sensors and digital twins	Sensors: 3–5; biological digital twins: 2–4	Sensor prototypes and computational models	Fluid stability, telemetry, data validation, privacy, and clinical workflow integration	Long-term *in vivo* sensor validation and prospective closed-loop rehabilitation trials

TRL, technology readiness level. TRL 1–2 indicates basic principles and early concepts; TRL 3–4 indicates experimental proof-of-concept and preclinical validation; TRL 5–6 indicates prototype validation in a relevant environment or early clinical feasibility; and TRL 7–9 indicates advanced clinical validation, regulatory maturity, or routine implementation. The TRL assignments in this review represent qualitative ACL-specific estimates derived from the maturity of available evidence and translational feasibility, rather than formal regulatory certification (U.S. Department of Health and Human Services, 2026).

## 3 Conclusion

The standard of care in ACL repair and reconstruction is approaching a critical inflection point, moving from passive mechanical substitution toward biologically informed and functionally integrated regeneration. As critically evaluated in this review, persistent clinical challenges—including ligament-bone interface stress concentration, delayed vascularization and ligamentization, proprioceptive denervation, and dysregulated postoperative inflammation—cannot be fully resolved by simply replacing one structural graft with another.

The successful translation of next-generation biointelligent ligament grafts requires a staged and clinically realistic approach. Cross-scale hierarchical biomaterials may help address the porosity-mechanics paradox; spatially retained release systems may reduce uncontrolled intra-articular diffusion and ectopic ossification; orthobiologics such as PRP and PRF may serve as feasible but still insufficiently standardized adjuncts; and piezoelectric or conductive strategies may eventually support proprioceptive neuralization. In parallel, implantable sensors, quantitative imaging, and digital twin concepts may shift postoperative management from generic time-based rehabilitation toward data-informed and patient-specific recovery.

Nevertheless, the field must avoid overextending laboratory concepts before demonstrating manufacturability, repeatability, cost-effectiveness, regulatory feasibility, and clinically meaningful benefit. The near-term translational priority is to integrate selected biointelligent functions into existing ACL surgical workflows, supported by large-animal validation, standardized orthobiologic protocols, quantitative imaging endpoints, and prospective clinical trials. By harmonizing biomimetic design, immune modulation, sensory restoration, and health-system feasibility, future ACL therapy may progress from structural reconstruction toward true neuro-mechanical regeneration.
